# Recruitment of ethnic minority patients to a cardiac rehabilitation trial: The Birmingham Rehabilitation Uptake Maximisation (BRUM) study [ISRCTN72884263]

**DOI:** 10.1186/1471-2288-5-18

**Published:** 2005-05-17

**Authors:** Kate Jolly, Gregory Y Lip, Rod S Taylor, Jonathan W Mant, Deirdre A Lane, Kaeng W Lee, Andrew J Stevens, the BRUM Steering Committee

**Affiliations:** 1 Department of Public Health and Epidemiology, University of Birmingham, Edgbaston, Birmingham B15 2TT, England; 2 University Department of Medicine, City Hospital, Birmingham B18 7QH, England; 3 Department of Primary Care & General Practice, University of Birmingham, Edgbaston, Birmingham B15 2TT, England

## Abstract

**Background:**

Concerns have been raised about low participation rates of people from minority ethnic groups in clinical trials. However, the evidence is unclear as many studies do not report the ethnicity of participants and there is insufficient information about the reasons for ineligibility by ethnic group. Where there are data, there remains the key question as to whether ethnic minorities more likely to be ineligible (e.g. due to language) or decline to participate. We have addressed these questions in relation to the Birmingham Rehabilitation Uptake Maximisation (BRUM) study, a randomized controlled trial (RCT) comparing a home-based with a hospital-based cardiac rehabilitation programme in a multi-ethnic population in the UK.

**Methods:**

Analysis of the ethnicity, age and sex of presenting and recruited subjects for a trial of cardiac rehabilitation in the West-Midlands, UK.

Participants: 1997 patients presenting post-myocardial infarction, percutaneous transluminal coronary angioplasty or coronary artery bypass graft surgery.

Data collected: exclusion rates, reasons for exclusion and reasons for declining to participate in the trial by ethnic group.

**Results:**

Significantly more patients of South Asian ethnicity were excluded (52% of 'South Asian' v 36% 'White European' and 36% 'Other', p < 0.001). This difference in eligibility was primarily due to exclusion on the basis of language (i.e. the inability to speak English or Punjabi). Of those eligible, similar proportions were recruited from the different ethnic groups (white, South Asian and other). There was a marked difference in eligibility between people of Indian, Pakistani or Bangladeshi origin.

**Conclusion:**

Once eligible for this trial, people from different ethnic groups were recruited in similar proportions. The reason for ineligibility in the BRUM study was the inability to support the range of minority languages.

## Background

Proportional representation of research participants by race is a requirement of The National Institutes of Health in the USA [1]. However, no such requirement exists in the UK, but concern has been expressed about the low representation of patients from ethnic minority groups in clinical trials [2,3]. There are some examples of good practice with a representative proportion of trial participants from minority ethnic groups recruited from areas of the UK with a diverse ethnic population [4,5]. In addition, there is some evidence that once patients have met the inclusion criteria for a clinical trial, then equal recruitment of patients from different ethnic groups can be achieved [6-8].

Much research on ethnicity has focused on testing hypotheses about differences in outcome by race or ethnicity whilst the issue that participants of trials should represent the population that will be receiving an intervention has been relatively neglected. This is essential for the generalisability of the results [8,9], and to achieve this, trial participants need to mirror the demographic profile of the disease group being studied. Whether participants in trials of chronic disease management reflect the population who are affected by such disease is an issue of growing importance in the UK as the black and minority ethnic population ages.

There are some unanswered questions with respect to the low representation of patients from minority ethnic groups in clinical trials. Firstly, compared to the majority white population, are patients from ethnic minority groups less likely to meet the eligibility criteria for a trial (e.g. because of language)? Secondly, are patients from ethnic minority groups more likely to refuse to participate in randomized trials [10]? Birmingham is an excellent place to explore this issue – some 30% of the population are from black and minority ethnic groups [11]. We have the opportunity to address these questions in relation to the Birmingham Rehabilitation Uptake Maximisation (BRUM) study, a randomized controlled trial (RCT) comparing a home-based with a hospital-based cardiac rehabilitation programme in a multi-ethnic population in the UK [12].

## Methods

The BRUM study recruited 525 (26.3%) participants from the 1997 patients who presented following a myocardial infarction, percutaneous transluminal coronary angioplasty (PTCA) or coronary artery bypass graft (CABG) surgery to one of four West Midland hospitals from February 2002–January 2004 [12]. The hospitals are located in an area of the UK with a high proportion of people of South Asian origin. The BRUM study sought to include patients who spoke either English or Punjabi (which is the most frequently spoken minority language locally), but was unable to include patients with other minority languages because of the lack of validated outcome measures in these languages.

Basic demographic data including self-defined ethnic group was sought on all presenting patients. Ethnic status was recorded using the same categories as the 2001 UK Census [13]. Data on ethnicity was obtained for 1933 (96.8%) of the patients presenting following a heart attack, PTCA or CABG, following which the ethnic categories were then combined into three broad groups: White, South Asian (Pakistani, Indian and Bangladeshi) and Other (Caribbean, African, Chinese). No-one described themselves as being of mixed ethnicity. Data on individual social class was not collected on all presenting patients, so postcodes were used to assign the Index of Multiple Deprivation 2004 (IMD) [14]. Details about the eligibility of patients, reasons for exclusion from the trial, and reason for declining to participate were collected. Data were entered into a Microsoft Windows ACCESS database (Microsoft Inc, USA) and analysed using SPSS version 12 (SPSS Inc, Chicago USA). Chi-squared tests were used to compare proportions across ethnic groups. For normally distributed data the one-way ANOVA was used to compare means, and the Kruskal-Wallis test to compare means for non-parametric data. Logistic regression was used to determine relative risks of eligibility and recruitment adjusted for sex, age, deprivation index and initial diagnosis.

## Results

Table 1 details the proportion of patients excluded and the reasons given. Reasons for non-recruitment are divided into exclusion criteria (e.g. language, severe disease, co-morbidity) and refusal. Significantly more South Asian patients were excluded (51.9%), but mainly for the reason of language (24.3%). Exclusions for co-morbidity were lowest in the South Asian patients (6.8% v 12.1% of white ethnic group), probably as a result of their significantly lower mean age (see table 1).

**Table 1:** Recruitment to and exclusion from the BRUM trial, by ethnic group

	**White**	**South Asian**	**Other**	**^f^p-value**
All presenting, n	1452	412	69	
MI n (%)	811 (56.4)	230 (56.5)	42 (61.8)	0.6
PTCA n (%)	514 (35.7)	149 (36.6)	19 (27.9)	
CABG n (%)	113 (7.9)	28 (6.9)	7 (10.3)	

Mean (SD) age	66.3 (12.0)	61.0 (12.4)	66.5 (12.2)	<0.001

Males n (%)	998 (68.9)	311 (75.5)	47 (69.1)	0.04

Mean (SD) Index of Multiple Deprivation (IMD) [14]	32.9 (17.1)	43.7 (16.6)	48.3 (16.8)	<0.001

Excluded n (%)	525 (36.1)	214 (51.9)	25 (36.2)	<0.001

Reason for exclusion (more than 1 may apply) n (%)				
^a^Language	4 (0.30)	120 (29.4)	3 (4.5)	<0.001
^b^High cardiac risk	191 (13.2)	50 (12.1)	6 (8.7)	0.7
^c^Comorbidity	175 (12.1)	28 (6.8)	6 (8.7)	0.01
In other trial	18 (1.2)	6 (1.5)	2 (2.9)	0.7
Out of area	76 (5.2)	18 (4.4)	2 (2.9)	0.4
^d^Sensory deficit	37 (2.5)	8 (1.9)	1 (1.4)	0.6
^e^Mental health	41 (2.8)	6 (1.5)	5 (7.2)	0.06
Deceased	19 (1.3)	5 (1.2)	0 (0)	0.6

Excluded for language only n (%)	2 (0.1)	100 (24.3)	3 (4.35)	<0.001

Eligible n (% of all presenting)	927 (63.8)	198 (48.1)	44 (63.8)	<0.001

Males (% of presenting males)	659 (65.7)	163 (52.4)	32 (68.0)	
Female (% presenting females)	268 (59.0)	35 (34.7)	12 (54.6)	
Mean (SD) IMD of eligible patients	32.3 (16.6)	41.2 (16.7)	47.1 (17.3)	<0.001

Refused (% of eligible)	514 (55.4)	109 (55.1)	27 (61.4)	0.2

Reason for refusal: n (%)				
Did not want rehabilitation	85 (16.5)	10 (9.2)	3 (11.1)	
Did not want to take part in research	272 (52.9)	38 (34.9)	11 (40.7)	
Preference for hospital programme	62 (12.1)	15 (13.8)	3 (11.1)	
No reason given	95 (18.5)	46 (42.2)	10 (37.0)	<0.001

Total recruited (% of eligible)	418 (45.1)	88 (44.4)	19 (43.2)	0.7
(% of all)	(28.8)	(21.4)	(21.3)	
Males (% of eligible males)	312 (47.3)	76 (46.6)	14 (43.8)	
Female (% of eligible females)	106 (39.6)	12 (34.3)	5 (41.7)	
Mean (SD) IMD of recruited patients	31.6 (15.7)	40.0 (16.9)	47.0 (15.2)	<0.001

^a^Language: unable to speak English or Punjabi, or Punjabi speaking nurse not available to recruit the patient.

^b^High risk: patients with risk factors excluding a home-based cardiac rehabilitation programme, e.g. severe angina, hart failure or documented arrhythmias.

^c^Comorbidity: physical conditions which would interfere with the ability to undertake a cardiac rehabilitation programme, e.g. severe arthritis, malignancy.

^d^Sensory deficit: severe hearing or visual loss such that patients would not be able to read written materials or communicate with research nurses on the telephone.

^e^Mental health: patients with case-note documented dementia or current mental illness.

^f^Chi square test (except for age comparison, oneway ANOVA; index of deprivation comparison, Kruskal-Wallis test)

Compared with the white ethnic group, the relative risk (95% CI) for eligibility was 0.42 (0.33, 30.55) for South Asians (table 2). However, there were no significant ethnic differences in the proportion of eligible patients recruited to the trial (45.1% of whites, 44.6% of South Asians, see Table 1). Compared with the British white ethnic group, the relative risk of recruitment of eligible patients was 0.59 (0.44, 0.78) for South Asians, when adjusted for age, gender, deprivation index and initial diagnosis.

**Table 2:** Eligibility and recruitment BRUM by ethnicity, adjusted for age, sex, deprivation and initial diagnosis

**Ethnic group**	**Adjusted RR^a ^of eligibility**	**95% CI**	**Adjusted RR of recruitment**	**95% CI**
White British	1.00 (base)		1.00 (base)	
Other White	1.52	0.81 to 2.84	01.10	0.60 to 2.02
South Asian	0.42*	0.33 to 0.55	0.59*	0.44 to 0.78
Afro-Caribbean	1.28	0.70 to 2.35	1.03	0.54 to 2.00
Other	0.86	0.16 to 4.19	0.97	0.23 to 4.21

White British used as the reference group (RR = 1.0)

^a^RR: relative risk

*P < 0.001

In each ethnic group (white, South Asian and other) women were less likely to be eligible for the study than men (table 1). This was statistically significant for the white and South Asian groups. Overall the women presenting with ischaemic heart disease were significantly older than the men (mean age 69.2 versus 63.6 years for men, p = 0.002) and were more likely to be excluded because of co-morbidity (16.8% v 8.6% of men, p < 0.001). Women of white and South Asian ethnicity were significantly less likely than the men of these ethnic groups to be recruited once eligible (p < 0.01) (table 1). There was no difference given in the reasons for declining to participate in the study by men and women.

The reasons for and rates of exclusion varied markedly between the three main South Asian groups, with exclusion rates of 29%, 63% and 70% among people of Indian, Pakistani and Bangladeshi origin respectively (Table 3). Figures for exclusions due to language barriers ranged from 5.1% (Indian) to 55% (Bangladeshi). In each South Asian sub-group the exclusion rate for language barriers was higher in people aged 65 years or more with 8.2% of people of Indian ethnicity, 43.6% of Pakistani and 87.5% of people of Bangladeshi ethnicity aged 65 years or more excluded because of language. This was approximately double the exclusion rates in people aged less than 65 years (see Table 3). A similar picture emerges for women, with higher exclusion rates for women in each of the South Asian sub-groups. Compared to people of white British origin, people of Pakistani and Bangladeshi origin were at a 4-fold increased risk of ineligibility to the trial, whilst people of Indian ethnicity were no more likely to be ineligible (see Table 3).

**Table 3:** Exclusions by ethnic group within the South Asian population presenting with a cardiovascular event

	**Indian** **n (%)** **n = 136**	**Pakistani** **n (%)** **n = 238**	**Bangladeshi** **n (%)** **n = 20**
Mean (SD) age, years	61.0 (12.6)	61.0 (12.4)	63.1 (7.3)

Males n, (%)	96 (70.6)	184 (77.3)	15 (75)

Mean (SD) IMD score	35.95 (15.8)	47.8 (15.7)	48.9 (14.4)

Any exclusion	40 (29.4)	151 (63.4)	14 (70)

Reason for exclusion (more than one may apply) n (%)			
^a^Language	10 (7.5)	94 (39.8)	11 (55)
^b^High cardiac risk	15 (11)	33 (13.9)	1 (5)
^c^Comorbidity	8 (5.9)	19 (8.0)	0 (0)

Excluded for language only:			1
All n (%)	7 (5.1)	78 (32.8)	1 (55)
Aged <65 years	3 (3.4)	34 (24.8)	4 (40)
65+	4 (8.2) p = 0.2	44 (43.6) p = 0.002	7 (87.5) p = 0.2
Gender males	4 (4.2)	51 (27.7)	6 (40)
females	3 (7.5) p = 0.4	27 (50) p = 0.003	5 (100) p = 0.04
Total recruited n	50	31	4
(% of eligible)	(52.1)	(35.6)	
(% of all presenting)	(36.8)	(13)	(20)

RR of ineligibility (95%CI)*	0.89 (0.6, 1.3)	4.0 (2.9, 5.5)	4.4 (1.6, 11.8)

^a^Language: unable to speak English or Punjabi, or Punjabi speaking nurse not available to recruit the patient.

^b^High risk: patients with risk factors excluding a home-based cardiac rehabilitation programme, e.g. severe angina, heart failure or documented arrhythmias.

^c^Comorbidity: physical conditions which would interfere with the ability to undertake a cardiac rehabilitation programme, e.g. severe arthritis, malignancy.

SD: standard deviation

*adjusted for age, sex and deprivation index using white British as the reference group (RR = 1.0)

## Discussion

This report has detailed the process of recruitment to a randomized controlled trial for patients from different ethnic groups from presentation with a cardiac event, through the eligibility criteria and consent process.

We have demonstrated that the point of inequality in recruitment between ethnic groups in this study occurred because of an inability to support the range of minority languages. This was despite additional measures employed to recruit Punjabi speaking patients, including a Punjabi speaking research nurse, translated (and recorded) patient information and the translation and validation of the Hospital Anxiety and Depression Scale into Punjabi. It is possible that those excluded on the grounds of language would have refused to participate in the trial, but we have no evidence to support or refute this possibility. Of those excluded as a result of language, 74% described themselves as of Pakistani origin and thus would have been catered for by the provision of Urdu and Mirapuri (an oral only language used by people of rural Pakistani origin). The socio-economic status of the presenting patients this is clearly a potential confounding factor, with people from minority ethnic groups who do not speak English being more likely to be of lower socio-economic status [15]. Presenting patients from minority ethnic groups had significantly higher deprivation indices than the white ethnic group, using the IMD as a measure of socio-economic status, in this study. Whilst the level of deprivation was a significant factor in the multiple regression analysis, ethnicity was still a significant factor in the likelihood of eligibility and recruitment to the trial when deprivation was included in the regression model.

We found no significant differences in the proportions of people from the three main ethnic groups who gave the reason for their refusing to participate as 'not wishing to take part in research.' This result differs from the evidence from the USA, which suggests that people from minority ethnic groups are less likely to agree to participate in clinical research because of a lack of trust [16]. The UK does not have the past history of the Tuskegee Syphilis study which appears to be the cause of this mistrust of research in black and minority ethnic groups in the US [17,18]. It is true that in this study a higher proportion of the minority ethnic groups who declined to participate in the trial gave no reason for their decision, so it is possible that there were differences in the reasons for not-participating that we failed to identify.

In addition, our findings provide further support for the limited evidence base that patients from ethnic minority groups can be recruited in a similar proportion to the majority population, once they have fulfilled the trial's eligibility criteria [7,8]. Other studies have tried to retrospectively estimate the proportion of patients from different ethnic groups, who would have been potential trial participants [19]. However, we believe that this is the first published report that has detailed the whole recruitment process of cardiac patients presenting with the index conditions, reasons for ineligibility and rates and reasons for refusal to participate. In the BRUM trial, the eligible patients of South Asian ethnicity were no more likely to decline to participate on the grounds of not wishing to take part in a research study than other ethnic groups.

## Conclusion

This research raises the issue of feasibility versus inclusivity. In this study, even with considerable effort, time and resources to try to recruit a representative population to the trial, we were unable to meet the wide language requirements. This must be considered in the context that this was a trial of a behavioural intervention with a psychological questionnaire as a main outcome measure. In other trials, with only objective clinical measurements as endpoints, it might be possible to exclude less patients due to language restrictions. In a geographical area of ethnic diversity, a number of languages would need to be supported to achieve equivalent recruitment rates for different ethnic groups, which has considerable cost and methodological implications. The importance of achieving a sample that is ethnically representative will depend on the research question and the context.

## Competing interests

The author(s) declare that they have no competing interests.

## Authors' contributions

All the authors were involved in the design and conduct of the trial. KJ undertook the analysis and drafted the paper. All the authors read and commented on the paper.

## Pre-publication history

The pre-publication history for this paper can be accessed here:


